# Genetic Restrictive Cardiomyopathy: Causes and Consequences—An Integrative Approach

**DOI:** 10.3390/ijms22020558

**Published:** 2021-01-08

**Authors:** Diana Cimiotti, Heidi Budde, Roua Hassoun, Kornelia Jaquet

**Affiliations:** 1 Department of Clinical Pharmacology, Ruhr-University Bochum, 44801 Bochum, Germany; 2 Experimental and Molecular Cardiology, St. Josef Hospital and BG Bergmannsheil, Clinics of the Ruhr-University Bochum, 44791 Bochum, Germany; heidi.budde@rub.de (H.B.); roua.hassoun@rub.de (R.H.)

**Keywords:** cardiomyopathy, restrictive cardiomyopathy, pediatric, sarcomere, contractile dysfunction, calcium, aggregation, gene expression

## Abstract

The sarcomere as the smallest contractile unit is prone to alterations in its functional, structural and associated proteins. Sarcomeric dysfunction leads to heart failure or cardiomyopathies like hypertrophic (HCM) or restrictive cardiomyopathy (RCM) etc. Genetic based RCM, a very rare but severe disease with a high mortality rate, might be induced by mutations in genes of non-sarcomeric, sarcomeric and sarcomere associated proteins. In this review, we discuss the functional effects in correlation to the phenotype and present an integrated model for the development of genetic RCM.

## 1. The Sarcomere

**Figure 1:**
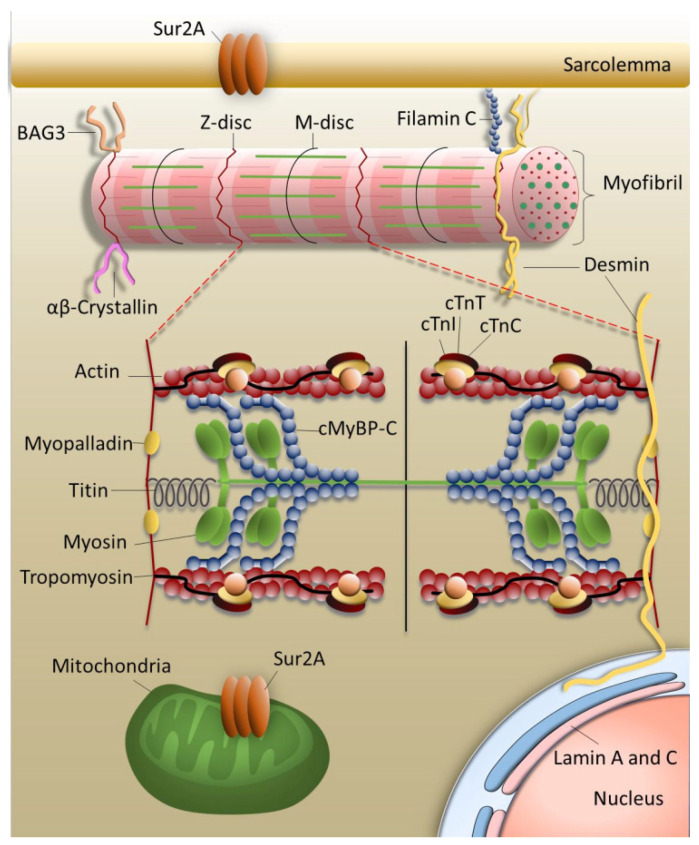
Organization of the contractile machinery. Cardiomyocytes are thickly packed with contractile elements, the myofibrils, which are connected to each other and via the cytoskeleton to the extracellular matrix and to the nucleus. Myofibrils are composed of sarcomeres, the smallest contractile units of the cardiac muscle cell. Proteins, whose genes are targets for mutations leading to restrictive cardiomyopathy are indicated. cMyBP-C = myosin binding protein C; cTnI, cTnT, cTnC = cardiac troponin subunits I, T and C; Sur2A = sulfonylurea receptor isoform 2A; BAG3 = Bcl2-associated athanogene 3. The figure was created using PowerPoint (Microsoft).

The sarcomere as the substructure of myofibrils is the contractile unit of striated muscles i.e. skeletal and cardiac muscle (Figure 1). It forms a symmetric unit with the M-disc in the center and is bordered by the Z-discs. M-disc and Z-disc provide the anchors for thin, thick and elastic filaments. The elastic filament is composed of the giant protein titin spanning half the sarcomere. The thin filament contains mainly filamentous actin and the regulatory proteins tropomyosin and troponin. The thick filament includes the motor protein myosin and the regulatory protein myosin binding protein C linking thick and thin filament. Ca^2+^-dependent interaction of myosin with actin leads to the gliding of the thin and thick filaments past each other, the sarcomere is shortened, myocytes and finally the whole muscle contract [1,2]. The sarcomere consists of a large number of highly organized proteins besides the proteins of thin and thick filaments and is linked to the extracellular space and nucleus via the cytoskeleton. In addition, a number of associated proteins connect the sarcomere to various signaling pathways. Such a complexity is indispensable to coordinate the contractile function of each cardiomyocyte and adapt the heart’s work continuously to the momentary demands of the body. Thus, the smooth work routine of all the sarcomeres within each cardiomyocyte of the heart muscle is pivotal for the contractile function of the heart and is based on the highly balanced interplay of sarcomeric proteins and of the sarcomeres themselves. Apparently, sarcomeric function is very sensitive to disturbances of any kind. Even one dysfunctional sarcomeric protein, altered protein-protein interactions, changes in sarcomeric structure and dynamics, alterations in expression, proteolytic degradation etc., progress to contractile dysfunctions and finally to cardiomyopathies and heart failure. Sometimes, compensation mechanisms to overcome the defects are provoked which, however, over time also may become pathological.

## 2. Cardiomyopathies

**Figure 2:**
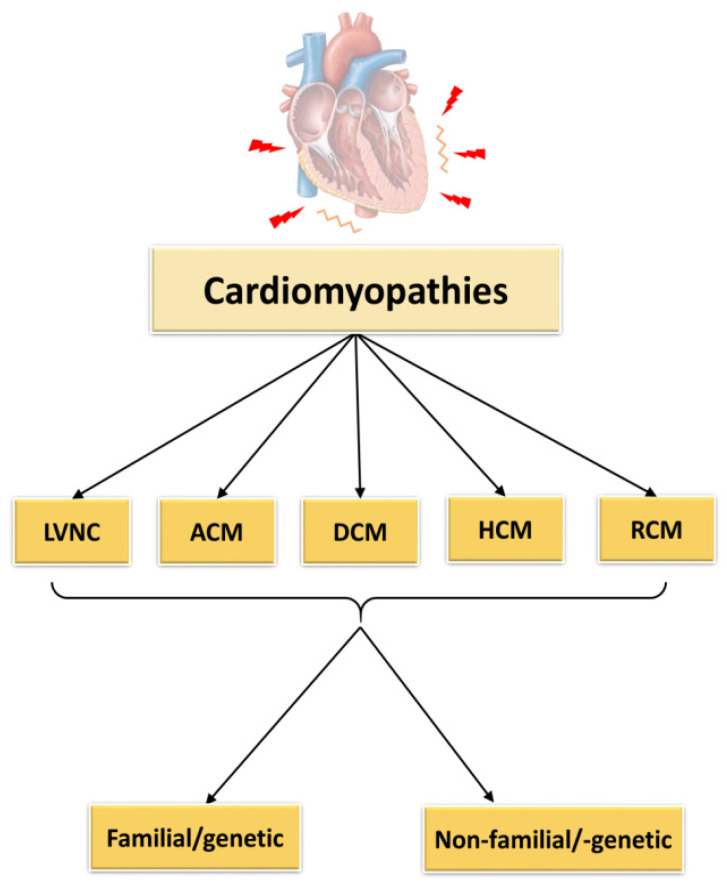
Classification of cardiomyopathies. LVNC = left ventricular non-compaction cardiomyopathy, ACM = arrhythmogenic cardiomyopathy, DCM = dilated cardiomyopathy, HCM = hypertrophic cardiomyopathy, RCM = restrictive cardiomyopathy. The figure was created using PowerPoint (Microsoft).

Cardiomyopathies are defined as cardiac diseases, in which the heart muscle is affected showing functional and structural defects [3]. According to the classification of cardiomyopathies as described by Elliott et al., 2008, they can be subdivided into RCM (restrictive cardiomyopathy), HCM (hypertrophic cardiomyopathy), DCM (dilated cardiomyopathy), ACM (arrhythmogenic cardiomyopathy) and unclassified cardiomyopathies as for example non-compaction cardiomyopathy (LVNC) (Figure 2). The causes of these cardiomyopathies may be genetic/familial or non-genetic and idiopathic.

### 2.1. Left Ventricular Non-Compaction Cardiomyopathy

LVNC seems to be the common cardiomyopathy type in the class of unclassified cardiomyopathies at least in children [4,5]. The origin of the disease is thought to lie in an impaired embryonic development, leading to a sponge-like ventricle and dilation due to abnormal trabeculations. In several cases mutations have been identified for example in the genes encoding a member of the dystrophin–related protein family, tafazzin, a mitochondrial membrane protein involved in cardiolipin metabolism, in dystrobrevin, or in a gene encoding lamin, located in the nuclear envelope [6]. But also sarcomeric genes as *MYH7*, *ACTC*, *TNNT2*, *TPM1*, *ZASP* are affected [7]. *TNNT2* seems to be involved in cardiogenesis in the regulation of atrial septal growth and formation of trabeculae [8]. However, the mechanism of disease development still is obscure and it is not clear, if and how these mutations impair correct embryonic development of the heart. Clinically, LVNC is associated with left ventricular dysfunction and severe arrhythmia, sudden cardiac death, or embolic stroke due to an enhanced risk of thrombus formation within the trabeculaes.

### 2.2. Arrhythmogenic Cardiomyopathy

ACM has an estimated frequency in the general population of 1:100 to 1:5000. Since sudden cardiac death similar to hypertrophic cardiomyopathy may occur as the first manifestation of the disease, there might be an additional number of unreported cases of ACM in the general population [9]. The hallmark of ACM is the replacement of ventricular cardiomyocytes by fibrotic and fatty tissue, which progresses with time. This might affect the right or left ventricle, or both, and finally leads to electrical instability and systolic dysfunction [10,11]. Like the other cardiomyopathies, ACM is genetically heterogeneous. Thus, for example, variants of proteins of the nuclear envelope like transmembrane protein 43 or lamin A/C have been correlated to ACM. The latter seems to induce mainly right ventricular and bi-ventricular cardiomyopathy [12,13]. In addition, mutations in *PLN* encoding phospholamban have been identified to cause ACM. In the Netherlands, the phospholamban p.R14del mutation has been declared a founder mutation responsible for the disease in 10–15% of all ACM patients [14]. Other ACM target genes encode the cardiac sodium channel and the sarcomeric protein titin [15,16,17]. Several rare single amino acid replacements in titin have been identified in different families (p.T8031C, p.A18579I + p.M33291T, p.A19309S, p.P308471L, p.T2896I). p.T2896I is located in the conserved Ig10 domain within the spring region of titin [16]. Also, nonsense filamin C variants have been correlated to either DCM or ACM. Recently, a filamin C intronic mutation was described in three Jewish families leading to reduced filamin C transcripts as well as aberrant filamin C protein variants [18]. Interestingly, these variants did not show a mislocalization of proteins such as glycogen synthase kinase-3β or plakoglobin considered typical for ACM [19]. Mainly, ACM is caused by mutations in genes encoding structural proteins as desmosomal proteins like plakoglobin, desmoplakin, plakophillin etc., with increased risk of sudden cardiac death and left ventricular dysfunction [20]. Desmosomes link desmin to the extracellular matrix. Dysfunctional desmosomes not only affect cell-cell communication, but also lead to cell death. Such a loss of cardiomyocytes is compensated finally by substitution with fat and/or fibrous tissue instead of new cardiomyocytes, since the regeneration capacity of cardiomyocytes is extremely low [21]. The disease usually manifests in adults or during adolescence. It is very rarely diagnosed in children, probably because of lacking symptoms. However, an early diagnosis would help to postpone manifestation of a severe ACM [22,23,24].

### 2.3. Dilated Cardiomyopathy

One of the common cardiomyopathies is DCM, which is mainly characterized by left ventricle dilation, systolic dysfunction and high morbidity. Main causes for DCM are infections, inflammation or toxins [25]. Also infants might be affected showing either mild or strong symptoms at diagnosis, but the disease onset in childhood is generally correlated with a high mortality rate. In a Swedish study, only 8% of the children recovered within the 25-year follow-up period [26]. Most of them had to undergo a heart transplantation, or a ventricular assist device or pacemaker was implanted, or they died before any of these options could be applied. An American study revealed that boys were more affected than girls and that also the ethnic origin seemed to play a role in disease progression [27]. Less frequently, DCM may also be caused by genetic defects, though there might be a significant number of undetected cases. Thus, according to Burkett & Hershberger, idiopathic DCM to about 50% is due to mutations [28]. The inheritance mainly is autosomal dominant, but also could be recessive, X-linked or even mitochondrial [29]. More than 60 genes have been associated to familial DCM [30,31]. The target genes for example might encode the sarcomeric proteins titin, cardiac troponin T (cTnT) and C (cTnC), actin, myosin heavy chain (MHC) or ion channels as the voltage gated sodium channel subunit alpha, as well as structural proteins e.g. lamin, filamin C, desmin [19,21]. One of the most prominent genes affected in familial DCM is *TTN* (up to 30% of all familial DCM cases up to date) encoding titin, the elastic filament of the sarcomere (Figure 1) [32]. Here, mostly truncation mutations have been observed in up to 25% of young DCM patients [33,34]. Most titin truncations in DCM patients occur in the A-band region of the sarcomere, whereby penetrance is clearly age dependent [35,36]. But also missense mutations have been described in *TTN* leading to similarly severe DCM as observed in patients with truncated titin. For example, Galan et al., 2020, recently showed that replacement of functional active cysteine residues in titin, whose oxidation affects titin stiffness and dynamics, leads to the development of DCM [37].

### 2.4. Hypertrophic Cardiomyopathy

The most frequent cardiomyopathy based on gene defects is HCM, whereby more than 1400 mutations in genes mainly encoding sarcomeric proteins have been identified up to date. Clinically, HCM is characterized by symmetrically or asymmetrically thickened heart walls, affecting in most cases the septum and/or the left ventricle. Diastolic dysfunction and a high risk of sudden cardiac death especially in young athletes are hallmarks in HCM. Histologically, cardiomyocytes appear enlarged and disarrayed and the cardiac muscle tissue shows fibrosis. In general, the disease manifestation is highly variable. Though there are also severe cases in young people, often the disease remains asymptomatic and thus undetected in the young [38,39,40]. The genes which are affected most in HCM patients are those encoding for myosin heavy chain (MHC) and cardiac myosin binding protein C (cMyBP-C). More than 50% of the reported HCM mutations have been detected in these two genes [41]. In *MYH7* (cardiac gene of MHC) and most other genes encoding sarcomeric proteins, predominantly missense mutations are found leading to single amino acid replacements in the resulting protein. In MHC, mostly amino acid replacements in the actin binding domain or the ATPase domain have been identified, affecting force production [42]. In case of cMyBP-C, mainly truncated proteins are formed, leading to haploinsufficiency [43]. On the molecular level, when using isolated recombinant protein fragments of MHC variants in functional assays, *MYH7* HCM mutations reduced force production. In contrast, in animal models and isolated variant MHCs enhanced contractility, i.e., increased and accelerated force production was observed [44]. The discrepancy of these observations may be due to effects of post-translational modifications or involvement of other proteins present in the more complex assay systems such as animal models and isolated whole cells or even myofibrils. Due to the complexity of the sarcomere and its interactions, reduced assay systems as reconstituted filaments are not able to reflect the situation in the sarcomere or even less in a cardiomyocyte or tissue; they just show dysfunction of the used proteins, but not necessarily the outcome in the cardiomyocyte or tissue. Thus, the enhancement of contractility fits to the generally observed increased Ca^2+^-sensitivity leading to enhanced activation at lower calcium concentrations than in healthy cardiac muscle [45,46]. Such a hypercontractility is known to lead to energy (ATP) depletion, but more importantly increase ADP- and decrease phosphocreatine levels, thereby affecting myosin cross bridges, force production and impairing re-extension [47,48].

### 2.5. Restrictive Cardiomyopathy

Restrictive cardiomyopathy (RCM) is a lethal, but rare disease which mostly is due to infiltration, and in a smaller percentage due to genetic disorders. In general, genetic RCM is characterized by near normal-sized left ventricle with enhanced stiffness and enlarged atria due to increased end-diastolic pressure in the ventricles. The disease is combined with an abnormal filling pattern and thus belongs to the diastolic diseases. Systolic function at least in the beginning of the disease is near normal but might be reduced at later stages of the disease. Sometimes also a mild hypertrophy is observed, making diagnostic distinction between RCM and HCM difficult [49].

The far most common cause of infiltrative diseases is amyloidosis that results from misfolding and deposition of proteins (amyloids) between the muscle fibers and/or within the walls of coronary arteries. The amyloids induce an enlargement of the heart walls giving an appearance of hypertrophy. However, the myofibers themselves are not affected as they are in HCM [50]. Two main types can be distinguished: the light chain (AL) and the transthyretin amyloidosis (ATTR). The latter type includes a hereditary sub-type caused by variants of the transthyretin protein, and a more common wild-type ATTR which is clearly age-related (“senile ATTR”) [51]. Similar as for DCM, the majority of idiopathic RCM cases are caused by gene defects, though up to date the knowledge on RCM genetics is still very poor [52,53]. In genetically based RCM, the inheritance usually is autosomal dominant. Genes with (non-infiltrative) RCM variants include also *TNNI3*, *TNNT2*, *TNNC1*, *TPM1*, *TTN*, *MYH7*, *MYL2*, *MYBPC3*, *MPN*, *DES*, *FLNC*, *LMNA*, *BAG3* (Table 1) and are similar to those of DCM, HCM and LVNC [52,54,55]. Most mutations have been identified in genes encoding for sarcomeric proteins, some in sarcomere associated proteins like small heatshock proteins such as crystallin αB, or their binding partners such as BAG3 — proteins whose dysfunction potentially leads to the accumulation of aggregated proteins (Table 1).

Several mutations in genes whose proteins are not directly involved in contractile function have been described in patients with RCM, among others desmin, filamin C and crystallin αB [56,57,58,59,60]. Usually, desmin mutations have been associated with DCM, however, a p.E413K mutation was found in a Polish family with a history of fatal heart diseases, in which 3 adult (30–60 year old) living members suffered of RCM [56]. Other family members also in young age suffered from heart disease and dies suddenly, but the diagnostic confirmation of RCM was not clear. The p.E413K desmin mutation is located in a conserved region involved in filament assembly which is different to the other mutations found in DCM patients. In patient muscle biopsies as well as in a cell culture model, desmin aggregates and disrupted Z-discs have been observed. Also, another desmin mutation which has been linked to RCM affects a splicing site within the *DES* gene and leads to disruption of the filamentous network of the cardiomyocytes [58]. In this case cardiac symptoms were diagnosed at the age of 46 in a Polish patient. More recently, a homozygous p.Y122H desmin mutation was identified in a RCM patient aged 19 [57]. This mutation is located within a region which is involved in the coiled coil formation of desmin dimers, and leads to abnormal cytoplasmic aggregation of desmin suggesting that this region may be a hotspot of cardiomyopathy-related mutations.

#### Pediatric RCM

Cardiomyopathies in children are overall rare, but often they are associated with a poor prognosis. The most common cardiomyopathies in children are DCM followed by HCM [27,97]. In children, genetically based RCM is seldom and accounts for less than 5% of the cardiomyopathy cases. Children with RCM show a rapid disease progression as well as a high mortality (50% survival within the first two years after diagnosis) [98,99]. Clinical characteristics are similar to RCM in adults, with diastolic dysfunction in absence of hypertrophy. In addition, pulmonary venous congestion, atrial fibrillation and SA block may occur, associated with an increased risk for arrhythmia and sudden cardiac death. [55,98]. Mogenson & Arbustini, 2009, suggested, that children with RCM exhibit a high risk for ischemia related events (infarcts, scarring, necrosis) even without signs of heart failure [100].

Several mutations have been detected in children with RCM affecting structural proteins, among which mutations in *FLNC* seem to be most prominent (Table 1). Filamin C cross-links actin filaments and is located at costameres, Z-discs and intercalated discs (Figure 1). The first RCM mutations in *FLNC* were described by Brodehl et al., 2016, in two different Canadian families leading to single amino acid replacements in conserved immunoglobulin domains, p.S1624L and p.I2160F [89]. Tissues of patients with p.S1624L showed filamin C aggregates and disrupted Z-discs. Members of this family became diseased at an age <10 years. In the family with p.I2160F, no aggregates were detected and the onset of the disease occurred much later. More recently two *de novo* mutations in *FLNC* have been found in children diagnosed with RCM at the age of 1, 3 and 15 years for the p.A1186V mutation and 6 months for the p.A1183L mutation [59]. Both mutations cause abnormal filamin C localization, disruption of Z-disks as well as aggregation. Similarly, a p.P209L mutation in *BAG3*, identified in 2018 in an eight year old boy diagnosed with myofibrillar myopathy and RCM, also caused aggregation of BAG3 and desmin, Z-disc abnormalities as well as dysregulated autophagy [96]. Though only relatively few mutations have been thoroughly characterized so far, myofibrillar disarray and protein aggregation seem to be common features in many mutations analyzed, supporting the idea of an infiltrative pathomechanism of RCM.

Other targets for RCM mutations are genes encoding sarcomeric proteins. In this group the main target is *TNNI3* encoding cardiac troponin I (cTnI), a sarcomeric regulatory protein [101]. *TNNI3* mutations were found to be predominant in pediatric RCM in a Chinese study [102]. Here, as well, detailed analyses of the underlying mechanisms are scarce, most suggesting contractile abnormalities such as increased Ca^2+^-sensitivity and impaired relaxation, which also occur in HCM. Interestingly, there seems to be a high rate of *de novo* infantile RCM mutations in the *TNNI3* gene, though a few *de novo* mutations have also been observed in *TTN* and *MYH7* [52,81,86,100].

*De novo* mutations as disease causing mutations are not easy to identify, especially in case of *missense* mutations leading to a single amino acid replacement [103]. Several factors have to be considered, as for example the localization of the mutation in a disease gene, the conserved position of the amino acid replacement and the function of the resulting protein, etc. A number of *de novo* mutations have been identified in pediatric cardiomyopathies. They come along with a very fast disease progression and a poor prognosis (Table 1). Pediatric RCM patients with *de novo* mutations frequently require a heart transplantation shortly after diagnosis to prevent premature death. Only a few of the known mutations, however, have been investigated on the mechanistic level.

## 3. Molecular Mechanisms in RCM

The knowledge on the molecular mechanism of primary cardiomyopathies is patchy, especially in RCM. In the beginning, there is just the mutation leading to an altered protein, which might be expressed at different levels than the wild type protein. When integrated into the sarcomere or into structures associated to the sarcomere, often the interplay with other sarcomeric/associated proteins is impaired, as well as protein and sarcomere dynamics, resulting in diastolic dysfunction, impaired structural stability, cell-to-cell communication and increased stiffness. Furthermore, specific signaling pathways are activated via associated proteins altering protein expression and degradation, survival, secretion of autocrine/paracrine hormones and exosomes, and thereby influence the performance not only of the heart, but also of other organs. The phenotype is the result of the whole network, including the genetic heterogeneity [104]. Therefore, a detailed knowledge on the molecular mechanisms of disease development is pivotal to develop specific therapeutic strategies. However, many of the mutations, especially those identified in RCM patients have just been described only in one family member, and further insights into the molecular mechanisms are lacking.

### 3.1. Contractile Dysfunction

#### 3.1.1. Calcium Signaling

**Figure 3:**
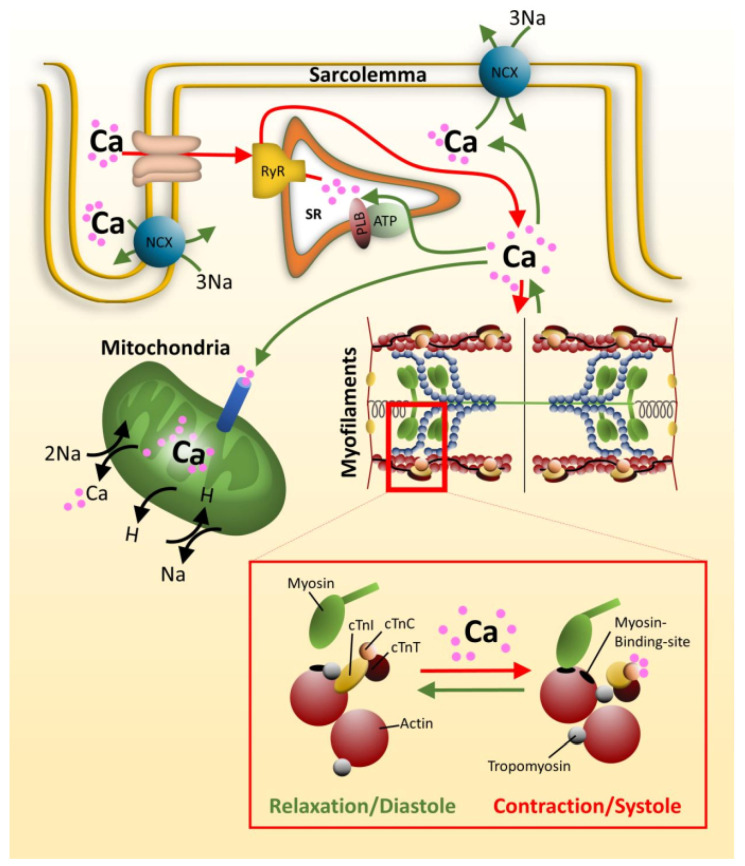
Role of Calcium in contractility. Voltage gated Ca^2+^-channels, located within T-tubuli, open upon depolarization of the sarcolemma. The resulting Ca^2+^-influx activates the nearby ryanodine receptors (RyR) of the sarcoplasmatic reticulum (SR), releasing Ca^2+^ from the SR. Ca^2+^ now binds to the Ca^2+^-sensor of the sarcomere, cardiac troponin C (cTnC), which forms together with cTnI and cTnT the troponin complex (see insert). Ca^2+^-saturation of cTnC triggers conformational changes within the troponin complex, releasing cTnI from actin. This enables tropomyosin to roll over on the actin filament to demask the myosin binding sites, and force production occurs. At the same time, Ca^2+^ is pumped back into the SR by a phospholamban (PLB) regulated SR Ca^2+^-ATPase, into the extracellular space by Na^+^/Ca^2+^ exchanger (NCX) and into the mitochondria. As the cytoplasmic Ca^2+^-concentration reduces, Ca^2+^ is released from cTnC, cTnI rebinds to actin and tropomyosin is forced back masking the myosin binding sites. The muscle relaxes. The figure was created using PowerPoint (Microsoft).

Calcium homeostasis in cardiomyocytes is important for many cellular processes as for example for the contractility of the cardiac muscle. A nervous impulse induces an action potential leading to a depolarization of the plasma membrane and opening of voltage gated calcium channels, the L-type Ca^2+^-channels located in the T-tubules of the cardiomyocytes. The influx of Ca^2+^ opens the ryanodine receptor integrated into the membrane of the sarcoplasmatic reticulum (SR), which forms the intracellular Ca^2+^-store. Ca^2+^ is released from the SR and triggers muscle contraction via binding to the Ca^2+^-sensor at the thin filament, namely cardiac troponin C (Figure 1 and Figure 3). For relaxation, there is a re-uptake of Ca^2+^ into the SR via SERCA, a Ca^2+^-ATPase. In addition, Ca^2+^ is pumped back into the extracellular space and into mitochondria.

Alterations in the Ca^2+^-handling are described as a main factor contributing to the development of arrhythmia or contractile dysfunction. The overall picture is that most DCM mutations are correlated with a reduced Ca^2+^-sensitivity of the filaments, in contrast most HCM and RCM mutations sensitize the sarcomeres for Ca^2+^, whereby the changes are more drastic in RCM than in HCM related mutations [61,105,106]. Increased myofilament Ca^2+^-sensitivity usually delays the relaxation process and leads to hypercontractility, enhanced energy consumption and enhanced risk for malignant arrhythmia [47,48,107]. An increased Ca^2+^-sensitivity of filaments was described for RCM mutations such as p.cTnI-A171T, -K178E, -D190G in permeabilized cardiomyocytes, and for p.cTnI-L144Q, -R145W, -A171T, -K178E and -R192H in skinned fibers [61,62]. These variants exhibited shorter resting sarcomere length, less inhibition capacity and developed less maximal tension. On the other hand, in rat cardiomyocytes some of the above mentioned variants showed no changes in Ca^2+^-sensitivity [62]. These discrepancies are probably dependent on the model system and assays used. Accordingly, the RCM mutations p.cTnI-R170G/W also exhibited a substantial increase in the Ca^2+^-sensitivity in skinned fibers from guinea pigs, but not in thin filament activation assays using reconstituted thin filaments [67]. Such discrepancies indicate that more proteins than the basic contractile proteins determine Ca^2+^-sensitivity. A more differentiated approach was provided by Sparrow et al., 2019, investigating two distinct HCM mutations, p.cTnT-R92Q and p.cTnI-R145G, in intact guinea pig cardiomyocytes using novel Ca^2+^-sensors [107]. Whereas p.cTnI-R145G directly increased the Ca^2+^-binding affinity of cTnC, p.cTnT-R92Q seems to increase the Ca^2+^-sensitivity via a cooperative mechanism. Both pathways finally impair relaxation. Davis et al., 2008, also described a shortened Ca^2+^-transient decay indicating impaired relaxation, which became more prominent upon higher pacing frequencies, implying an effect on Ca^2+^-homeostasis of the cardiomyocytes [62]. An increase in Ca^2+^-sensitivity is observed not only with most HCM and RCM inducing troponin variants but also with pathogenic variants of other sarcomeric thin and thick filament proteins, e.g., myosin light chain, tropomyosin, myosin heavy chain, myosin binding protein C [108]. In RCM, the Ca^2+^-sensitizing effect is usually much more prominent than in HCM. However, while HCM patients are at a high risk for malignant arrhythmia and sudden cardiac death (SCD), in RCM patients the risk for SCD is not higher than in HCM patients and is often due to ischemia [109]. Thus, altered Ca^2+^ handling is not the only key factor leading to arrhythmia in HCM. On the other hand, the prominent Ca^2+^ sensitizing effects in RCM might substantially contribute to the more pronounced ventricle stiffness [47]. Exceptional ventricle stiffness together with increased diastolic pressures, a hallmark of RCM, may cause in children ischemia and fatal arrhythmia [110,111].

#### 3.1.2. Cardiac Troponin I and the Interplay of Sarcomeric Proteins in RCM

Cardiac troponin I (cTnI) is the inhibitory subunit of the heterotrimeric troponin complex (cTn), the main regulatory protein of the thin filament. cTnI binds to the Ca^2+^ binding subunit cTnC and the tropomyosin binding subunit cTnT within the troponin complex and forms a molecular switch. Under relaxing conditions, i.e. during diastole, cTnI strongly binds to actin and tropomyosin and thus blocks the interaction of the myosin motor domain with actin, thereby inhibiting muscle contraction. Upon Ca^2+^-binding to cTnC, cTnI is released from actin/tropomyosin which allows an azimuthal movement of tropomyosin on the actin filament. The binding sites for the myosin heads are demasked and force production and contraction are enabled. Furthermore, as we recently reported, there is a direct interaction between cardiac troponin and cardiac myosin binding protein C (cMyBP-C), the regulatory protein of the thick filament which connects thick and thin filament [67]. This interaction implies a coordinated action of the two regulatory proteins, troponin and cMyBP-C in the regulation of muscle contraction. In addition to that, regulation of muscle contraction is under hormonal control leading to phosphorylation of sarcomeric proteins, thereby fine-tuning contractile function. Thus, phosphorylation of titin modulates passive tension and altered phosphorylation affects Ca^2+^-sensitivity and force development [104,112]. cTn and cMyBP-C both are phosphorylated by protein kinase A (PKA) upon ß-adrenergic stimulation. In cardiac disease, the phosphorylation patterns and their effects within the cardiomyocyte may change [113]. Furthermore, pathogenic protein variants may exhibit altered phosphorylation. For example, PKA phosphorylation of p.cTnI-R145G, a HCM variant, is suppressed thus uncoupling ß-adrenergic modulation via cTn [114] Likewise, PKA phosphorylation of p.cTNI-R145W, a RCM variant, leads to uncoupling of the response however not by itself, but through altered phosphorylation of additional proteins as titin and cMyBP-C [115]. Additionally, proteins involved in calcium homeostasis are regulated by phosphorylation, such as the L-type calcium channels, ryanodine receptors and phospholamban, the regulator of SERCA activity, as well as cardiac troponin I at the sarcomere. PKA-dependent phosphorylation on the one hand enhances the Ca^2+^-influx into the sarcoplasm and thereby promotes contraction, and on the other hand accelerates relaxation. These relationships underline that a balanced phosphorylation is a prerequisite for contractile function and that alterations in phosphorylation and/or phosphorylation responses as observed in RCM and other cardiomyopathies lead to contractile dysfunction [116].

**Figure 4:**
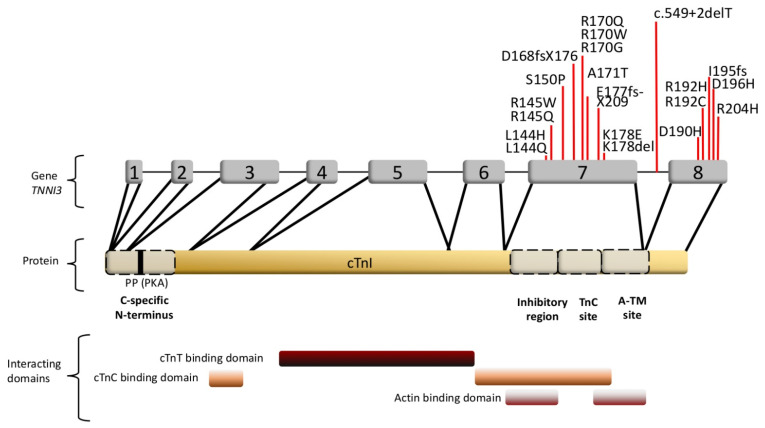
cTnI organization. Exons, RCM mutations and binding sites are indicated. A-TM: a second actin-tropomyosin binding site, PP (PKA): Serines 22, 23 phosphorylated by the proteinkinase A (PKA). The RCM mutations mainly affect exon 7 and 8, encoding for the inhibitory region, TnC binding domains and the A-TM site. The figure was created using PowerPoint (Microsoft).

**Figure 5:**
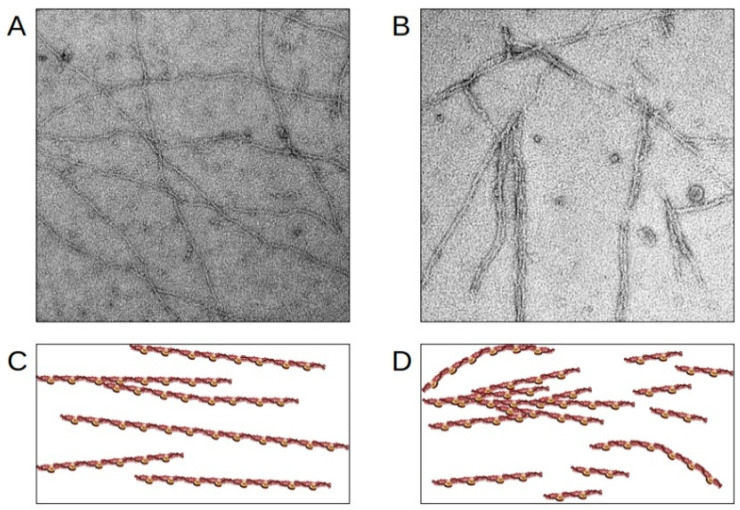
Disruption of thin filaments induced by RCM cTnI-variants. (**A**,**B**) Electron microscopic images obtained with reconstituted cardiac thin filaments. (**C**,**D**) schematic presentation. (**A**,**C**) wild-type cTnI; (**B**,**D**) the RCM cTnI-variant p.R170W leads to shortened, wavy and partially aggregated thin filaments. The figure was created using PowerPoint (Microsoft).

The cardiac troponin I gene is the main target for RCM mutations in the sarcomere [52]. The mutations, mostly leading to single amino acid replacements, are concentrated in cTnI in the regulatory C-terminal regions of the protein (Table 1, Figure 4) [117]. There are two actin binding regions in the cTnI C-terminus: the inhibitory domain (aa130–150) together with the switch domain (aa151–167), and the mobile region (aa168–210). The inhibitory domain blocks the myosin-actin interaction in the absence of calcium, the switch domain binds to the N-terminal domain of cTnC during muscle activation, and the mobile C-terminus is only bound to actin/tropomyosin in the relaxed, i.e. resting state of the muscle [118,119]. The clustering of RCM mutations in the regulatory C-terminus implies that interactions of cTnI with actin/tropomyosin/cMyBP-C and dynamics might play a pivotal role in the contractile dysfunction in cardiomyopathies and contribute to disturbed sarcomere stability and integrity. In addition, it has been shown for some RCM mutations in *TNNI3* that the incorporation of the variant cTnIs into the thin filament is impaired. According to Davis et al., 2008, who used a rat and rabbit gene transfer model and isolated cardiomyocytes for their investigations, mutants located in the cTnI inhibitory domain such as p.cTnI-L144Q and p.cTnI-R145W are integrated into the thin filament to a lower extent than the wild type cTnI [62]. In this case, no change in Ca^2+^-sensitivity was observed and PKA dependent Ca^2+^-sensitization was not affected. Thus, individuals carrying these mutations have a low amount of mutant cTnI incorporated in their sarcomeres, but nevertheless show a highly disordered relaxation. In accordance, we recently described that p.TnI-R170W, which is located in the mobile C-terminal domain of cTnI, was incorporated into reconstituted thin filaments to a lower degree and showed a reduced binding affinity towards actin and an increased affinity towards tropomyosin [67]. In addition to that, the binding affinity of this cTnI variant for cMyBP-C was increased. Only in skinned fibers, but not in reconstituted thin filaments, a massive Ca^2+^-sensitization and reduction of the cooperative communication along the thin filament was detected due to this mutation. Moreover, a decreased thin filament stability was detected using electron microscopic imaging and reconstituted thin filaments. Reconstituted thin filaments containing p.R170W showed a significantly higher amount of filament breaks, clustering of filaments and wavy instead of straight filaments (Figure 5). Thus, it appears that pathogenic cTnI variants impair the interplay between sarcomeric proteins leading to contractile dysfunction and structural instability, which may contribute to arrhythmia. Furthermore, reduced incorporation of mutant proteins might lead to an excess of free protein, potentially leading to aggregation in the cytosol.

### 3.2. Protein Aggregation

A hallmark for non-sarcomeric RCM is protein aggregation, a problem known for non-sarcomeric familial RCM as amyloidosis or glycogen storage disease, where aggregates are deposited between the myofibrils [50]. In sarcomere-related RCM, the p.Pro209Leu mutation in the *BAG3* gene leads to early-onset RCM and myofibrillar myopathy with typical intra-sarcoplasmic bodies composed of BAG3 protein and desmin aggregates [96]. Alterations of the myofibril integrity and Z-disc structures, as well as an impaired autophagy were also observed. BAG proteins bind to the Hsp70 ATPase domain and thereby inhibit activity of these chaperones. The BAG3/Hsp70 complex controls protein aggregation and may be also in complex with small heatshock proteins as crystallin αB (sHsp5) [120,121]. In the cardiac muscle, crystallin αB is mainly located in the I band of the sarcomere associated to titin’s N2BA region, and in the Z-disc associated with desmin, actin and α-actinin, preventing aggregation of these proteins and stabilizing the myofibrils [122]. Brodehl et al., 2017, found the first crystallin αB mutation in patients with RCM leading to a dysfunctional crystallin αB and sarcoplasmatic aggregates [60]. The formation of aggregates seems also a problem in RCM causing filamin C (*FLNC*) and desmin (*DES)* mutations [57,89]. Pathogenic mutations in the genes of crystallin αB and desmin leading to aggregate formations have been linked to an impaired protein quality control and autophagy [123]. In addition, HCM mutations in cMyBP-C knock-in mice showed an impairment of the ubiquitinylation/proteasome system [88,124]. Similar investigations for sarcomeric RCM mutants are missing. However, it is tempting to speculate that diastolic dysfunction and/or protein aggregation due to an impaired protein quality control system (protein degradation, autophagy or expression) both pivotally contribute to the RCM phenotype.

### 3.3. Gene Expression and Mosaicism

According to Salman et al., 2018, there is a substantial amount of pathogenic intronic mutations, namely about 10%, which may alter splicing mechanisms [125]. Thereby, insertions or deletions occur which may alter the reading frame and induce a premature stop codon leading to a truncated protein. This has been observed e.g. for cMyBP-C HCM mutations in a study of 400 Italian patients [126]. In addition to single nucleotide variants, larger gene copy number variations (CNVs) can also lead to loss-of-function mutations due to exon skipping, premature stop or duplication of exons. CNVs have been identified in patients with congenital heart disease, HCM, DCM, LVNC and ACM, but only few studies included RCM [127,128,129]. In a study by Ceyhan-Birsoy et al., next generation sequencing-based CNV analysis of a large cardiomyopathy patient cohort revealed no clinically significant CNVs in any of the 25 RCM patients included [130]. Due to the relatively low number of cases analyzed and the generally rare occurrence of CNVs, the involvement of CNVs in RCM cannot be excluded, though.

Truncation of proteins is often caused by frameshift mutations within exons. It is widely accepted for sarcomeric proteins that truncations cause haploinsufficiency, whereas missense mutations are integrated into the sarcomere. Thus, truncated cMyBP-C HCM mutants are expressed in a ratio of 1:5, wild type protein versus mutant, according to Helms et al., 2014 [131]. It is thought that on mRNA level mutants are formed in the same amount as wild type. However, there seems to be an imbalance in the translation of the mutant versus wild type protein, which has been described by Helms et al., 2014, for several HCM mutations, with protein levels being highly dependent on the type of mutation. Similar results have been published by Tripathi et al., 2011, who determined the amount of various myosin heavy chain HCM mutants in soleus muscle and cardiac muscle [132]. They found reduced amounts of mutant protein to ca. 30–60% of the wild type protein dependent on the mutation. mRNA transcripts were unaffected indicating that either translation is downregulated by specific micro-RNAs (miRs) or long non-coding RNAs (lncRNAs), or the resulting proteins are unstable and disposed. On the other hand, Montag et al., 2018, showed that also mRNA levels could vary; they determined different levels of mutant mRNA as well as protein for ß-MHC p.G716R [133]. The mutant mRNA was predominant with 89%, while protein was only present to 29.9%. The higher the imbalance, the earlier the disease will onset and the worse is the prognosis [132]. Similar observations have been made also for other sarcomeric protein mutants as cMyBP-C [134]. Unfortunately, no similar studies have been performed for RCM mutations up to date, but it is plausible that this allelic imbalance is not a typical feature of HCM, but also occurs in other cardiomyopathies. In a mouse model, where different transgenic mice strains expressed the RCM mutant p.cTnI-R192H at different levels, Li et al., 2013, described a dose-dependency of diastolic dysfunction, stiffness and premature death [135]. Severity, mortality and early onset increased with increased levels of mutant protein. Thus, for pediatric RCM one would assume that mutant to wild type ratios would differ largely due to the early onset and severity. This imbalance in protein expression could result in an imbalance of sarcomeric contractile function and contribute to reduced sarcomeric stability. In fact, we observed breaks and clustering of reconstituted thin filaments with the infantile RCM mutations p.cTnI-R170G/W, though this might not be typical for RCM only [67]. Moreover, the pattern of cardiomyocytes showing different amounts of mutant and wild type proteins might contribute to the dysfunction of the myocardium [136,137]. In different cardiomyocytes of a tissue mRNAs are formed in different amounts at random, leading to different expression levels of wild type and HCM mutant proteins. Such an imbalance not only might be responsible for myocyte disarray, but also be the reason for asynchronous contraction (increased risk of lethal arrhythmia), enhanced stretch stress with subsequent activation of specific pathways as the fibrotic pathways, death pathways etc. Similar problems might also occur in other cardiomyopathy types.

## 4. Problems and Prospects in RCM

### 4.1. Diversity and Diagnostic Disparity of Cardiomyopathies

Many novel mutations have been detected in the last years and have been correlated to cardiomyopathies, and in most cases pathogenicity classification was performed according to the ACMG guidelines, though the strength of evidence is not always fully reported [138]. Furthermore, obtaining strong evidence for pathogenicity is often difficult due to small family sizes, incomplete segregation analysis and study of family members, as well as lack of mechanistic investigations, nicely reviewed by Burke et al., 2016 [139].

The boundaries especially between HCM and RCM are not clear [140]. There is also an overlap with LVNC and even DCM since many sarcomeric disease genes are identical for RCM, HCM, DCM and LVNC. Thus, one might assume that the positions of the mutations within the affected genes differ in the various cardiomyopathy types. However, this often is not the case. Thus, with a few exceptions most of the HCM mutations and the few known DCM mutations in *TNNI3* are also clustered in the regulatory C-terminus of the resulting cTnI protein [141]. Whereas the p.Y122H and p.E413K desmin RCM mutations are located in a different domain than the DCM mutations, as discussed above. Furthermore, different mutations at the same position in a gene may lead to different cardiomyopathy types. For example, mutations in *TNNI3* lead to HCM or RCM, the p.R145G replacement in cTnI induced HCM, the p.R145W replacement RCM [52,142]. Members of one family may even develop a different cardiomyopathy type despite carrying the same mutation as was described for p.cTnI-R145W in a large Korean family [143]. Some members developed HCM, others RCM, or even no disease phenotype. Nevertheless, p.cTnI-R145W is classified as a RCM inducing mutation and was first described by Mogensen et al., 2003 [52]. On the other hand, up to date amino acid replacements at the position R170 in the mobile C-terminus of cTnI seem to be linked exclusively to RCM. p.R170G/W lead to infantile severe RCM, and also the p.R170Q exchange, identified in a South African family, was classified as an RCM mutant with mild focal hypertrophy and early onset [63,67]. In another earlier study by Kaski et al., 2009, however, p.cTnI-R170Q was identified in children with HCM with an early onset (<13 y) [68]. Thus, it remains unclear, if the clinical classification into HCM and RCM is unambiguous, since sometimes also RCM patients develop a mild hypertrophy.

Such a diversity in mutation-cardiomyopathy correlations complicates a phenotype prediction based on a mutation. Surely, the location of the amino acid replacement within the affected protein is important for the development of a cardiomyopathy. For example, arginine 170 is located in the second actin/tropomyosin binding site of the regulatory C-terminus of cTnI. A reduced affinity to this binding site, which is determined by the type of amino acid exchange, reduces the inhibitory capacity of cTnI, facilitates the interaction of the cTnI switch region with cTnC [67]. This interaction again affects the calcium affinity of cTnC and calcium sensitivity of the actin-myosin interaction, promoting contraction and impairing relaxation and affecting energy consumption. Subsequently, the Ca^2+^-homeostasis in the cell is disturbed affecting signaling cascades. The resulting contractile dysfunction might impair the structural integrity of sarcomeres and, in concert with altered protein expression and altered interactions with associated proteins as well as altered post-translational modifications, might pivotally contribute to the specific phenotype. In addition, it is thought that modifiers as sex hormones, polymorphisms in other genes (exons and introns) or even other mutations also contribute to the disease phenotype [125,144,145]. A specific feature of RCM is protein aggregation. In case of protein aggregates due to sarcomeric or sarcomere associated protein variants, sarcomere structure is disrupted leading to contractile dysfunction and stiffness.

### 4.2. Development of the RCM Phenotype: An Integrated Approach

**Figure 6:**
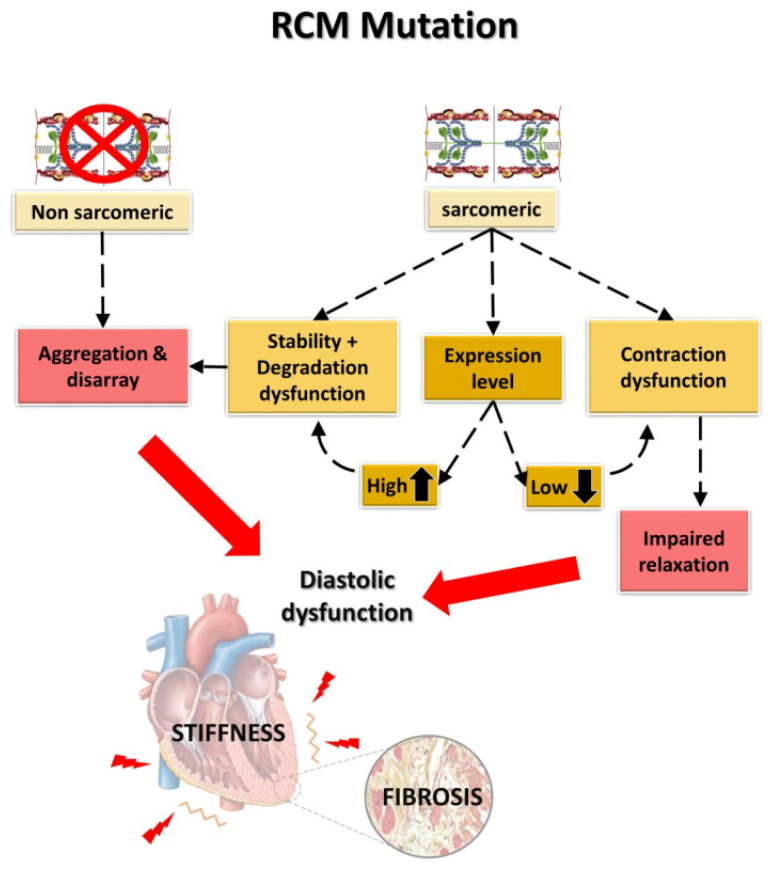
Factors leading to the development of the genetic RCM. While non-sarcomeric mutations lead to aggregations and promote cytoskeletal and sarcomeric disarray, sarcomeric mutations may affect the cardiac function in different ways, depending on their expression levels, stability, tendency to aggregate and to induce contractile dysfunction. Both finally lead to diastolic dysfunction and increased stiffness. The figure was created using PowerPoint (Microsoft).

In summary, a clear differentiation of RCM and HCM based on the underlying mutations and clinical diagnosis is still very difficult. There is a strong need for further detailed mechanistic studies and broad genetic testing to obtain a better understanding of genotype-phenotype correlations. Because of the many overlapping characteristics and of familial HCM and RCM phenotypes within the same family, Burke et al., 2016, proposed that RCM might be a special, more severe form of HCM, but not an independent cardiomyopathy [139]. However, there are distinct clinical specificities of RCM, as for example the extraordinary stiffness of ventricles and pulmonary hypertension due to the high pressure in the ventricles. Some molecular features seem to be promising to explain the characteristic RCM phenotype, such as protein expression, allelic imbalance, aggregation phenomena connected with impaired protein quality control, and sarcomeric dysfunction including diastolic dysfunction due to increased Ca^2+^-sensitivity and ADP accumulation. Taking all what is known into consideration, RCM might develop in two ways (Figure 6): one starts from mutant sarcomeric protein with or without altered protein expression in comparison to the wild type protein. A lower mutant protein level might induce haploinsufficiency, inducing contractile dysfunction via impaired sarcomeric dynamics, sarcomeric structure and inter- and extra-sarcomeric protein-protein interactions. Contractile dysfunction including disturbed structural integrity of the sarcomere can also be induced by the mutation itself. An increased protein expression or protein stability might lead to an excess of mutant protein which has to be degraded. Modifiers, as for example additional intronic polymorphisms or mutations which might affect gene expression and/or alterations in the interaction with components of the protein control system, might lead to protein aggregations disrupting sarcomeric structure and inducing contractile dysfunction. All these features might finally result in enhanced ventricle stiffness and a higher risk of malignant arrhythmia. Cell-to-cell imbalance could further amplify the effects. The other pathway involves mutations in non-sarcomeric proteins, leading to protein aggregates between myofibers which may also affect contractile function, increase stiffness and the risk of malignant arrhythmia. A detailed understanding of these correlations would be helpful to develop specific therapies which are missing up to date.

### 4.3. Therapeutic Options

The current treatment of genetic RCM mainly follows general heart failure management, e.g., ACE inhibitors, ß-blockers, Ca^2+^-desensitizers and anticoagulation therapy. In a progressed state of the disease, a left ventricular assist device is often used to bridge the time gap to heart transplantation. Thus, no specific therapy exists for RCM. Therapeutic options based on the RCM model described here could additionally include targeting translation of mutant proteins via miRs or lncRNAs, or targeting contractile function using for example calcium desensitizers as epigallocatechin or ranolazin, as proposed by Mosqueira et al., 2018, which promotes Ca^2+^-efflux via NCX and counteracts reduced blood supply, thus inhibiting malignant arrhythmia [146]. In this study, several HCM mutations in the *MYH7* gene were investigated using isogenic pluripotent stem cell-derived cardiomyocytes to study their resultant properties. The authors found energy depletion to be a core factor for hypertrophy and also identified several changes in protein and RNA expression by transcriptomics. They further propose gene modifiers and lncRNAs as therapeutic targets. There are few more examples that targeting miRs or lncRNAs might be successful, too [147,148]. In DCM, fibrosis is a major problem. Zhang et al., 2019, found that miR132 is reduced in cardiomyocytes of DCM rat models [149]. miR132 mainly regulates the *PTEN* gene. Upregulation of miR132 or silenced *PTEN* repressed fibrosis via the PI3/Akt pathway. Up to date, no such studies exist for RCM therapy using lncRNA or miRs/siRNA, and only few studies concerning the effects of the calcium desensitizer epigallocatechin. Zhang et al., 2015, showed in a restrictive mouse model that epigallocatechin could restore diastolic function [150]. Thus, the sarcomere is also a promising target for drugs. Especially for pediatric RCM, it would be helpful at least to postpone the time point for heart transplantation.

As formation of protein aggregates also seems to be an important feature in RCM, similar therapeutic approaches as implemented for e.g. amyloidosis may also be conceivable for treatment of RCM. Here, the formation of aggregates could be reduced or prevented e.g. by tetramere stabilization with Tafamidis or Diflunisal, or by disruption of oligomeres again by epigallocatechin or by doxycycline and taurosodeoxycholic acid [151]. Furthermore, the use of monoclonal antibodies directed against the aggregated protein might be an option, as recently proposed by Popkova et al., 2020, for the treatment of amyloidosis [152]. A stimulation of ubiquitinylation and HDAC6 for the disposal of aggregates in autophagosomes or lysosomes is also conceivable [153,154].

## 5. Conclusions

Cardiomyopathies, a complex of cardiac diseases classified into five main forms, result from inherited and acquired causes. Inherited cardiomyopathies are caused by mutations often, but not exclusively, located in genes encoding for sarcomeric proteins. While many studies have been conducted on the more common HCM and DCM, data on the rarer RCM remain scarce. Though RCM has distinctive clinical features such as increased myocardial stiffness and atrial dilation, in many cases the phenotype is not clear or shows features of e.g. both HCM and RCM, making proper classification difficult.

On the molecular level, there seem to be two particular mechanisms involved in RCM, which can occur alone or in conjunction. On the one hand, strongly increased myofilament Ca^2+^-sensitivity and altered protein-protein interactions of the contractile proteins are the most common functional alterations observed in inherited RCM. On the other hand, infiltrative processes including aggregation and impaired protein quality control have mostly been described for mutations in non-sarcomeric proteins, but no such studies have been performed with sarcomeric proteins. Moreover, additional factors apart from the genetic mutation can influence the severity, prognosis, and the clinical phenotype of the disease. So far, it is not clear which mechanism (or combination of mechanisms) finally determines the manifestation of RCM vs. other cardiomyopathy phenotypes.

Further mechanistic studies are thus crucial for a deeper understanding of the phenotype-genotype linkage. Furthermore, they could provide the basis for the development of specific therapeutic approaches for RCM, taking into account both the contractile as well as the infiltrative mechanisms.

Additionally, other genes and variants than those known today might also be associated with RCM. Thus, genetic testing of RCM patients with a probably heritable disease and relatives should generally be performed, especially in the young, as well as of patients with idiopathic RCM or in cases of unexplained cardiac death. In familial cases, the greatest possible coverage of the family by genetic testing might help to uncover additional variants modifying the manifestation of different phenotypes among family members. Definitely, still more data is needed to understand RCM on the genetic and mechanistic level.

## Figures and Tables

**Table 1:** Overview of mutations in sarcomeric and non-sarcomeric proteins associated with restrictive cardiomyopathy.

Gene/Protein	Variant	Allele Origin	No. of Affected Families (No. Mutation Carriers)	Age at Presentation/Onset (Youngest Patient)	Molecular Effects/Remarks	Ref. #
*TNNI3*/cTnI	p.L144Q	unknown	-	17 years	Myofibrillar disarray, Ca^2+^-sensitization + incr. basal force, red. maximal force (in reconstituted filaments), low ATPase inhibition + maximal activity, decreased incorporation in thin filaments	[52,61,62]
	p.L144H	familial	1(3)	20–30 years	mild hypertrophy	[63]
	p.R145W	familial	2(2)	19 years	Low ATPase inhibition, Ca^2+^-sensitization (reconstituted filaments), decreased incorporation in thin filaments	[61,62,64]
	p.R145Q	unknown	-	9 years	Occurred in combination with R192C	[64]
	p.S150P	familial	1(3)		unknown	[65]
	c.549+2delT	*De novo*	-	<1 year	truncation	[64]
	p.D168fsX176	unknown	1(1)	23 years	unknown	[66]
	p.R170G	*De novo*	-	3 years	Ca^2+^-sensitization (skinned fibers), thin filament instability, impaired interaction with cMyBP-C and Tpm	[55,67]
	p.R170W	*De novo*	-	8 months	Ca^2+^-sensitization (skinned fibers), thin filament instability, impaired cMyBP-C interaction, decreased incorporation in thin filaments	[55,67]
	p.R170Q	*De novo*	-	15 years	Unknown; also associated with HCM in another study	[63,68]
	p.A171T	unknown	-	63 years	Mild Ca^2+^-sensitization	[52,61]
	p.E177fsX209	*De novo*	-	6 years	disarray	[54]
	p.K178E	*De novo*	-	3 years	Low ATPase inhibition, Ca^2+^-sensitization (skinned fibers), increased max. ATPase activity	[52,61]
	p.K178del	*De novo*	-	<11 years	unknown	[64]
	p.D190H	familial	1(13)	11 years	Ca^2+^-sensitization (*in-vitro* ATPase activity), red. cooperativity	[52]
	p.R192C	unknown	-	9 years	Occurred in combination with R145Q	[64]
	p.R192H	*De novo*	-	16 years	Ca^2+^-sensitization, red. cooperativity (*in-vitro* ATPase activity), impaired relaxation, disarray, fibrosis (mouse model), increased incorporation in thin filaments	[52,61]
	p.I195fs	*De novo*	-	24 years	Mild fibrosis and hypertrophy, no aggregation	[69]
	p.D196H	familial	1(10)	41 years	3 members homozygous, heterozygous relatives asymptomatic; fibrosis, mild hypertrophy, no deposits	[70]
	p.R204H	*De novo*	-	14 years; 3 years	unknown	[71,72]
*TNNT2*/cTnT	p.I79N	familial	1(9)	53 years	No disarray (biopsy); fibrosis; Ca^2+^-sensitization (mice, skinned fibers); also associated with HCM in other studies	[73,74]
	p.E96del	*De novo*	-	1 year	Disarray, fibrosis (biopsy); Ca^2+^-sensitization (skinned fibers, *in-vitro* ATPase activity); impaired inhibition (ATPase), impaired relaxation (skinned fibers), with fetal TnI the effects are less severe	[75,76]
	p.E136K	familial	1(3)	3.5 years	Myocyte vacuolation, no disarray	[54]
*TNNC1*/cTnC	p.A8V and p.D145E	familial	1(4)	8 months	Compound heterozygosity; HCM to RCM transition; Ca^2+^-sensitization (fibers), slow Ca^2+^_off rate, impaired relaxation	[77,78,79]
*TPM1*/Tpm	p.E62Q and p.M281T	familial	1(11)	9 years	Compound heterozygosity; disruption of sarcomeres (biopsy), reduced Ca^2+^ transient amplitudes (HL-1 cells)	[80]
*TTN*/Titin	p.Y7621C	familial	1(5)	12 years	Fibrosis, myofilament degradation, Z-disk distortion	[81]
*MYH7*/MHC	p.Y386C	*De novo*	-	9 months	Mild fibrosis, no disarray (biopsy)	[82]
	p.R721K	familial	1(1)	43 years	Together with p.Sur2A-R1186Q; arrhythmia	[83]
	13bp del	*De novo*	-	49 years	unknown	[84]
	p.G768R	familial	1(2)	15 months	No tissue abnormalities (biopsy), no disarray or fibrosis	[85]
	p.838L	*De novo*	-	5 months	Mild disarray, no infiltration (biopsy), arrhythmia	[86]
*MYBPC3*/cMyBP-C	p.Q463X	familial	1(3)	34 years	unknown	[87]
	p.E334K	*De novo*	-	45 years	Increased polyubiquitinylation and degradation (cell model)	[87,88]
*DES*/desmin	p.Y122H	familial	1(1)	19 years	Homozygous; impaired intermediate filament assembly, desmin aggregates (iPSC)	[57]
	735+1G>T	familial	1(1)	46 years	Myopathy, alternate splicing	[58]
	p.E413K	familial	1(3)	30 years	Desmin aggregation, granulofilamentous deposits (biopsy); disruption of intermediate filaments, aggregation (cell model)	[56]
*FLNC*/filamin C	p.A1183L	*De novo*	-	6 months	perinuclear aggregates (zebrafish skeletal muscle)	[59]
	p.A1186V	*De novo*	-	1.4 years	Absence of filamin C and desmin in intercalated discs, Z-line streaming (biopsy); perinuclear aggregates (zebrafish skeletal muscle)	[59]
	p.S1624L	familial	1(4)	3 years	Aggregates, Z-disk disorganization, impaired desmin localization (biopsy, cell model)	[89]
	p.G2151S	familial	1 (2)	15 years	Sarcomere disorganization, Filamin C deposits (biopsy)	[90]
	p.I2160F	familial	1 (6)	15 years	disrupted Z-disks, impaired desmin localization (biopsy, cell model)	[89]
	p.V2297M	familial	1 (5)	44 years	Impaired Filamin C association with sarcomeres (biopsy), reduced contractility (ESC model)	[91]
	p.P2298L	familial	1 (8)	3 years	No deposits, no sarcomeric disarray (biopsy); perinuclear Filamin C and actin aggregation (cell model)	[92]
	p.P2301L	*De novo*	-	41 years	Significant fibrosis, no deposits (biopsy)	[90]
	p.Y2563C	*De novo*	-	1 year	Regular sarcomeric structure, no deposits (biopsy); randomly distributed Filamin C and actin aggregates (cell model)	[92]
*MYPN*/myopalladin	p.Q529X	familial	1 (3)	7 years	Truncated variant without NEBL and a-actinin binding domains; nuclear deformations, myofibrillar degeneration, impaired a-actinin localization, CARP aggregation, Z-disk disruption (biopsy, cell model); nuclear enrichment of truncated Mypn, impaired MAPK signaling, down-regulation of CARP (mice)	[93,94]
*LMNA*/Lamin A	p.E279RfsX201	familial	1(2)	53 years	No infiltration (biopsy)	[95]
*CRYAB*/crystallin αB	p.D109G	familial	1(2)	19 years	Protein aggregation, Z-disk disruption (biopsy, cell model)	[60]
*BAG3*/Bag3	p.P209L	unknown	-	8 years	Aggregation of BAG3 and desmin, Z-disk alterations, myofibrillar disarray, undegraded autophagosomes, increased autophagy regulators (biopsy)	[96]
*ABCC9*/Sur2A	p.R1186Q	familial	1(1)	43 years	Together with p.MHC-R721K; arrhythmia	[83]
